# Type 2 Diabetes Risk Allele Loci in the Qatari Population

**DOI:** 10.1371/journal.pone.0156834

**Published:** 2016-07-06

**Authors:** Sarah L. O’Beirne, Jacqueline Salit, Juan L. Rodriguez-Flores, Michelle R. Staudt, Charbel Abi Khalil, Khalid A. Fakhro, Amal Robay, Monica D. Ramstetter, Iman K. Al-Azwani, Joel A. Malek, Mahmoud Zirie, Amin Jayyousi, Ramin Badii, Ajayeb Al-Nabet Al-Marri, Maria J. Chiuchiolo, Alya Al-Shakaki, Omar Chidiac, Maey Gharbiah, Abdulbari Bener, Dora Stadler, Neil R. Hackett, Jason G. Mezey, Ronald G. Crystal

**Affiliations:** 1 Department of Genetic Medicine, Weill Cornell Medical College, New York, New York, United States of America; 2 Division of Pulmonary and Critical Care Medicine, Department of Medicine, Weill Cornell Medical College, New York, New York, United States of America; 3 Department of Genetic Medicine, Weill Cornell Medical College-Qatar, Doha, Qatar; 4 Division of Translational Medicine, Sidra Medical Research Centre, Doha, Qatar; 5 Department of Biological Statistics and Computational Biology, Cornell University, Ithaca, New York, United States of America; 6 Department of Medicine, Hamad Medical Corporation, Doha, Qatar; 7 Laboratory Medicine and Pathology, Hamad Medical Corporation, Doha, Qatar; 8 Department of Medical Statistics and Epidemiology, Hamad Medical Corporation, Doha, Qatar; 9 Department of Medicine, Weill Cornell Medical College-Qatar, Doha, Qatar; Tor Vergata University of Rome, ITALY

## Abstract

**Background:**

The prevalence of type 2 diabetes (T2D) is increasing in the Middle East. However, the genetic risk factors for T2D in the Middle Eastern populations are not known, as the majority of studies of genetic risk for T2D are in Europeans and Asians.

**Methods:**

All subjects were ≥3 generation Qataris. Cases with T2D (n = 1,124) and controls (n = 590) were randomly recruited and assigned to the 3 known Qatari genetic subpopulations [Bedouin (Q1), Persian/South Asian (Q2) and African (Q3)]. Subjects underwent genotyping for 37 single nucleotide polymorphisms (SNPs) in 29 genes known to be associated with T2D in Europeans and/or Asian populations, and an additional 27 tag SNPs related to these susceptibility loci. Pre-study power analysis suggested that with the known incidence of T2D in adult Qataris (22%), the study population size would be sufficient to detect significant differences if the SNPs were risk factors among Qataris, assuming that the odds ratio (OR) for T2D SNPs in Qatari’s is greater than or equal to the SNP with highest known OR in other populations.

**Results:**

Haplotype analysis demonstrated that Qatari haplotypes in the region of known T2D risk alleles in Q1 and Q2 genetic subpopulations were similar to European haplotypes. After Benjamini-Hochberg adjustment for multiple testing, only two SNPs (rs7903146 and rs4506565), both associated with transcription factor 7-like 2 (TCF7L2), achieved statistical significance in the whole study population. When T2D subjects and control subjects were assigned to the known 3 Qatari subpopulations, and analyzed individually and with the Q1 and Q2 genetic subpopulations combined, one of these SNPs (rs4506565) was also significant in the admixed group. No other SNPs associated with T2D in all Qataris or individual genetic subpopulations.

**Conclusions:**

With the caveats of the power analysis, the European/Asian T2D SNPs do not contribute significantly to the high prevalence of T2D in the Qatari population, suggesting that the genetic risks for T2D are likely different in Qataris compared to Europeans and Asians.

## Introduction

Susceptibility to type 2 diabetes (T2D), a disorder characterized by chronic hyperglycemia in the context of inadequate insulin secretion and peripheral tissue insulin resistance, is influenced by inheritance with superimposed dietary and lifestyle factors [1, 2]. Genome-wide association studies (GWAS) and exome sequencing have identified a large number of genes linked to susceptibility to T2D, helping to understand the pathogenesis of T2D and the genes that influence pancreatic beta-cell function/insulin secretion and insulin resistance [3, 4].

The majority of genetic studies of T2D have been carried out in populations of European descent, populations where the prevalence of T2D in adults is 2.4 to 14.7% [3–7]. In contrast, other than studies of candidate genes, little attention has been focused on the genetic risk factors associated with Middle Eastern populations, despite the growing epidemic of T2D in the Middle East, with an estimated prevalence of 6 to 23.9% in adults [7].

In the context of the high prevalence of T2D in the Middle East, we initiated a study of genetic risk factors of T2D among Qataris, a highly consanguineous population located at the migration crossroads of Africa and Eurasia with an adult prevalence of T2D of 22% [7]. The Qatari population is comprised of 3 genetic subgroups, Bedouin (Q1), Persian/South Asian (Q2) and African (Q3), all of which have a similar, high prevalence of T2D [8, 9]. To assess whether the European and/or Asian risk alleles explained, in part, the high prevalence of T2D in the Qatari population, we evaluated 1,124 Qataris with T2D and 590 Qatari controls with 37 SNPs representing 29 genes for which there are definitive links to an increased risk for T2D in European and Asian populations [3, 6, 10]. Based on analysis of Qatari haplotypes in regions flanking known T2D risk single nucleotide polymorphisms (SNPs), demonstrating similarity of the Q1 and Q2 Qatari subpopulations to European haplotypes, our power analysis predicted that, with this high prevalence of T2D, if these variants contribute to the high prevalence of T2D in Qataris, the European and Asian T2D at risk SNPs should be easily detectable in this Qatari population. Contrary to our hypothesis, we found that risk allele frequencies for almost all of these T2D SNPs were similar in Qatari and European populations, with only 2 of 37 SNPs associated with T2D in the European or Asian populations significantly associated with T2D in a high prevalence Qatari population, suggesting that the genetic risks for T2D in Qataris differ from those of the European and Asian populations.

## Methods

### Ethics Statement

Subjects were recruited from Hamad Medical Corporation (HMC) clinics and written informed consent obtained under protocols approved by the Joint Institutional Review Boards (JIRB) of Hamad Medical College and Weill Cornell Medical College Qatar (WCMC-Q).

### Study Population

A total of 1,714 subjects (1,124 cases with T2D and 590 controls) were enrolled. T2D was diagnosed based on the American Diabetes Association (ADA) criteria, consisting of fasting plasma glucose ≥ 126 mg/dL, 2 hr plasma glucose ≥200 mg/dL during an oral glucose tolerance test, and/or HbA1C ≥ 6.5% [11].

A questionnaire, physical examination and medical record review were performed and blood collected on each subject. Demographic data including age, date of birth and gender, personal and family history of diabetes was recorded along with self-reported history of gestational diabetes, duration of diabetes and medication use including duration of insulin therapy if applicable. Physical characteristics such as height and weight were collected and body mass index (BMI) calculated. Laboratory tests including HbA1C, and blood glucose levels were performed.

All subjects were over the age of 30 and were a minimum three generations Qatari. Cases were excluded if any of the following were present: history of type 1 diabetes, maturity onset diabetes of the young (MODY), maternally inherited diabetes and deafness syndrome (MIDD), a first degree relative with type 1 diabetes, or secondary diabetes.

### Haplotype Analysis

To test if previously described European and Asian T2D SNPs were potentially relevant to the Qatari population, European, Asian, African, and Qatari haplotypes were compared in the region of the chosen 37 T2D risk alleles [3, 6, 10] and an additional 27 tag SNPs with high minor allele frequency adjacent to these loci (S1 Table). The haplotypes of 100 Qataris who had undergone deep sequencing (detailed below), were inferred and divided into intervals of 2,000 SNPs. With the 1,000 Genomes Project Phase 1 as a reference, haplotype blocks in the regions flanking these 64 SNPs in Europeans, Africans and Asians were compared to Qatari subpopulation haplotype blocks for each of the three major genetic subgroups in the Qatari population: Bedouin (Q1), Persian/South Asian (Q2) and African (Q3) [8, 9]. The population with most similar haplotype was determined using SupportMix [12], by inferring the percentage of Qatari haplotypes assigned to European, Asian, African, or Admixed for each subpopulation.

### Qatari Genetic Subpopulation Genotyping

DNA was extracted from blood using the QIAamp DNA Blood Maxi Kit (Qiagen Sciences Inc, Germantown, MD). The 1,714 subjects were classified into the three genetic subgroups described in the Qatari population [8, 9] using a TaqMan SNP Genotyping Assay (Life Technologies, Carlsbad, CA) for a previously described panel of 48 ancestry informative SNPs [8, 9]. Average genotype call rate was 96% and analyzed in STRUCTURE with K = 3. Q1, Q2 or Q3 population was assigned if highest proportion was >65%; otherwise they were classed as “admixed” (S1 Fig).

### T2D SNP Genotyping

Allele frequencies were determined for T2D cases (n = 1,124) and controls (n = 590) for 21 SNPs associated with T2D by GWAS in various populations representing 16 genes, and an additional 27 tag SNPs adjacent to these loci (Table 1 and S1 Table). A subgroup (n = 626 cases, and n = 326 controls) of the total study population underwent genotyping for an additional 16 T2D associated SNPs, representing 15 genes (Table 1 and S1 Table). All genotyping was performed using TaqMan SNP Genotyping Assays (Life Technologies, Carlsbad, CA) as per manufacturer’s protocols. The 29 genes were classified into those associated with insulin resistance or insulin secretion [3, 6, 10] (S1 Table).

**Table 1:** Type 2 Diabetes Risk Allele SNPs.

Category	Gene	SNP	All Qatari			Q1 +Q2^1^			Q1^1^			Q2^1^			Q3^1^			Admixed		
			p value^2^	OR^3^	CI^4^	p value^2^	OR^3^	CI^4^	p value^2^	OR^3^	CI^4^	p value^2^	OR^3^	CI^4^	p value^2^	OR^3^	CI^4^	p value^2^	OR^3^	CI^4^
Beta-cell	CAMK1D/CDC123	rs12779790	5.0x10^-1^	1.13	0.909–1.401	2.5x10^-1^	1.26	0.992–1.603	5.5x10^-1^	1.33	0.972–1.812	8.0x10^-1^	1.16	0.792–1.685	9.7x10^-1^	0.45	0.174–1.176	9.5x10^-1^	0.85	0.442–1.631
dysfunction	CDKAL1	rs10946398	4.3x10^-1^	1.13	0.931–1.364	5.5x10^-1^	1.12	0.905–1.394	9.9x10^-1^	1.01	0.764–1.340	4.8x10^-1^	1.32	0.935–1.859	9.7x10^-1^	1.50	0.713–3.158	9.5x10^-1^	1.23	0.704–2.158
		rs7756992	8.0x10^-1^	1.06	0.840–1.327	5.8x10^-1^	1.13	0.865–1.469	9.9x10^-1^	1.01	0.718–1.420	4.8x10^-1^	1.45	0.919–2.278	9.7x10^-1^	1.14	0.485–2.672	9.5x10^-1^	0.71	0.355–1.411
	CDKN2A-B	rs564398	7.6x10^-1^	0.94	0.772–1.134	5.6x10^-1^	0.90	0.727–1.107	6.4x10^-1^	0.87	0.651–1.151	9.1x10^-1^	0.94	0.689–1.293	9.7x10^-1^	1.33	0.418–4.224	9.5x10^-1^	1.08	0.599–1.961
		rs10811661	1.9x10^-1^	0.81	0.651–1.002	3.3x10^-1^	0.82	0.645–1.039	5.5x10^-1^	0.82	0.595–1.125	7.8x10^-1^	0.82	0.567–1.176	7.4x10^-1^	0.18	0.049–0.683	9.5x10^-1^	0.98	0.531–1.804
	G6PC2	rs560887	9.7x10^-1^	1.01	0.752–1.305	6.7x10^-1^	0.90	0.819–1.515	8.4x10^-1^	0.89	0.767–1.640	9.1x10^-1^	1.11	0.510–1.596	9.7x10^-1^	0.79	0.358–4.474	9.5x10^-1^	1.67	0.246–1.440
	HHEX/IDE/KIF11	rs1111875	1.8x10^-1^	0.82	0.685–0.979	1.8x10^-1^	0.79	0.649–0.968	9.0x10^-1^	0.94	0.719–1.232	2.1x10^-1^	0.63	0.465–0.859	9.7x10^-1^	1.29	0.614–2.725	9.5x10^-1^	0.75	0.443–1.269
	HNF1A	rs7957197	2.7x10^-1^	0.80	0.611–1.058	4.0x10^-1^	0.81	0.589–1.101	6.9x10^-1^	0.83	0.550–1.240	8.6x10^-1^	0.85	0.505–1.427	9.7x10^-1^	1.72	0.488–6.040	9.5x10^-1^	0.67	0.294–1.516
	HNF1B (TCF2)	rs4430796	1.8x10^-1^	0.80	0.649–0.991	1.8x10^-1^	0.75	0.595–0.953	5.0x10^-1^	0.71	0.521–0.962	7.8x10^-1^	0.83	0.567–1.223	9.7x10^-1^	1.21	0.422–3.445	9.5x10^-1^	0.84	0.430–1.639
	JAZF1	rs864745	1.4x10^-1^	0.81	0.686–0.957	2.4x10^-1^	0.83	0.691–0.999	9.6x10^-1^	0.96	0.755–1.230	2.1x10^-1^	0.68	0.514–0.909	9.7x10^-1^	0.69	0.308–1.546	9.5x10^-1^	0.92	0.560–1.508
	KCNJ11	rs5215	8.1x10^-1^	1.04	0.853–1.258	7.5x10^-1^	1.04	0.841–1.293	9.0x10^-1^	1.07	0.794–1.447	9.4x10^-1^	1.04	0.762–1.431	9.7x10^-1^	0.60	0.173–2.061	9.5x10^-1^	1.05	0.615–1.779
	MADD	rs7944584	8.0x10^-1^	0.94	0.724–1.224	7.5x10^-1^	1.06	0.792–1.421	5.5x10^-1^	1.37	0.934–2.008	7.8x10^-1^	0.75	0.463–1.223	9.7x10^-1^	0.23	0.045–1.220	5.9x10^-1^	0.52	0.249–1.089
	NOTCH2	rs10923931	7.9x10^-1^	1.08	0.825–1.399	5.8x10^-1^	1.16	0.846–1.597	9.9x10^-1^	1.01	0.646–1.563	6.9x10^-1^	1.35	0.847–2.143	9.7x10^-1^	0.96	0.453–2.050	9.5x10^-1^	1.06	0.506–2.222
	THADA	rs7578597	7.6x10^-1^	0.90	0.667–1.207	5.6x10^-1^	0.83	0.570–1.197	9.0x10^-1^	0.87	0.465–1.620	8.0x10^-1^	0.84	0.527–1.325	9.7x10^-1^	1.50	0.693–3.244	9.5x10^-1^	0.95	0.462–1.952
Reduced insulin	ADRA2A	rs10885122	2.4x10^-1^	0.82	0.642–1.036	4.0x10^-1^	0.82	0.620–1.089	5.5x10^-1^	0.73	0.514–1.042	1.0x10^0^	1.00	0.609–1.640	9.7x10^-1^	0.96	0.415–2.232	9.5x10^-1^	0.68	0.345–1.327
secretion	FADS1	rs174550	1.9x10^-1^	1.26	0.998–1.585	1.8x10^-1^	1.33	1.026–1.710	5.5x10^-1^	1.28	0.935–1.757	4.8x10^-1^	1.44	0.918–2.262	9.7x10^-1^	0.94	0.214–4.097	9.5x10^-1^	1.11	0.558–2.220
	GCK	rs1799884	9.4x10^-1^	1.02	0.802–1.287	7.1x10^-1^	0.93	0.715–1.214	9.6x10^-1^	1.04	0.742–1.444	6.9x10^-1^	0.74	0.461–1.182	9.7x10^-1^	1.06	0.358–3.150	9.5x10^-1^	1.35	0.642–2.840
		rs4607517	9.8x10^-1^	1.00	0.786–1.265	6.4x10^-1^	0.91	0.695–1.179	9.9x10^-1^	1.00	0.720–1.395	6.8x10^-1^	0.72	0.450–1.151	9.7x10^-1^	1.61	0.434–5.992	9.5x10^-1^	1.32	0.616–2.808
	GLIS3	rs7034200	2.7x10^-1^	0.83	0.668–1.042	5.8x10^-1^	0.89	0.689–1.141	5.5x10^-1^	0.81	0.585–1.116	9.0x10^-1^	1.11	0.726–1.697	9.7x10^-1^	0.59	0.209–1.691	6.3x10^-1^	0.58	0.303–1.107
	LGR5/TSPAN8	rs7961581	6.5x10^-1^	1.07	0.913–1.258	5.8x10^-1^	1.09	0.909–1.296	5.5x10^-1^	1.19	0.943–1.507	9.7x10^-1^	0.97	0.740–1.283	9.7x10^-1^	1.33	0.614–2.876	9.5x10^-1^	1.02	0.633–1.632
	PROX1	rs340874	8.1x10^-1^	1.04	0.843–1.281	5.8x10^-1^	1.11	0.877–1.414	5.5x10^-1^	1.23	0.901–1.669	9.0x10^-1^	1.11	0.736–1.665	9.7x10^-1^	0.71	0.233–2.164	9.5x10^-1^	0.82	0.470–1.416
	SLC2A2 (GLUT2)	rs11920090	3.3x10^-1^	1.24	0.922–1.657	3.5x10^-1^	1.31	0.933–1.829	5.5x10^-1^	1.40	0.909–2.165	9.1x10^-1^	1.11	0.651–1.891	9.7x10^-1^	2.41	0.719–8.059	9.3x10^-1^	0.54	0.220–1.348
	SLC30A8	rs11558471	2.7x10^-1^	0.78	0.581–1.058	6.8x10^-1^	0.89	0.637–1.252	9.9x10^-1^	1.02	0.643–1.632	8.0x10^-1^	0.80	0.470–1.367	9.7x10^-1^	0.50	0.133–1.902	5.9x10^-1^	0.43	0.171–1.096
		rs13266634	9.6x10^-1^	1.02	0.738–1.396	6.9x10^-1^	1.11	0.769–1.614	9.6x10^-1^	1.07	0.590–1.942	8.1x10^-1^	1.20	0.737–1.936	9.7x10^-1^	0.21	0.044–1.005	9.5x10^-1^	1.06	0.488–2.298
	TCF7L2	rs4506565	**3.7x10**^**-2**^	1.33	1.121–1.586	1.8x10^-1^	1.24	1.023–1.507	5.5x10^-1^	1.18	0.916–1.515	4.8x10^-1^	1.34	0.986–1.820	9.7x10^-1^	1.35	0.637–2.875	**4.5x10**^**-2**^	2.43	1.409–4.195
		rs7901695	1.8x10^-1^	1.20	1.016–1.414	3.6x10^-1^	1.15	0.958–1.389	7.6x10^-1^	1.11	0.866–1.412	6.8x10^-1^	1.22	0.908–1.620	9.7x10^-1^	0.95	0.473–1.916	1.2x10^-1^	1.90	1.155–3.131
		rs7903146	**2.9x10**^**-2**^	1.36	1.146–1.618	1.8x10^-1^	1.28	1.057–1.551	5.5x10^-1^	1.20	0.932–1.537	3.4x10^-1^	1.40	1.034–1.890	9.7x10^-1^	1.18	0.503–2.779	7.4x10^-2^	2.17	1.291–3.649
Pancreatic	HHEX	rs5015480	1.9x10^-1^	0.86	0.731–1.007	1.8x10^-1^	0.82	0.685–0.981	8.0x10^-1^	0.92	0.728–1.167	2.1x10^-1^	0.69	0.521–0.915	9.7x10^-1^	1.07	0.541–2.116	9.5x10^-1^	1.90	1.155–3.131
development	IGF2BP2	rs4402960	4.8x10^-1^	1.11	0.933–1.312	2.6x10^-1^	1.20	0.985–1.451	5.0x10^-1^	1.32	1.025–1.711	9.4x10^-1^	1.04	0.769–1.414	9.7x10^-1^	0.99	0.482–2.030	9.5x10^-1^	0.83	0.523–1.328
Insulin	GCKR	rs780094	1.8x10^-1^	1.25	0.647–0.993	4.0x10^-1^	1.18	0.671–1.078	5.5x10^-1^	1.32	0.563–1.024	9.1x10^-1^	0.91	0.725–1.655	9.7x10^-1^	2.22	0.156–1.316	9.5x10^-1^	1.19	0.424–1.657
resistance	KLF14	rs972283	9.4x10^-1^	1.02	0.808–1.278	6.8x10^-1^	0.92	0.705–1.193	9.0x10^-1^	0.93	0.666–1.298	8.6x10^-1^	0.87	0.549–1.368	9.7x10^-1^	1.77	0.629–5.005	9.5x10^-1^	1.26	0.639–2.469
	PPARG	rs1801282	8.3x10^-1^	0.96	0.685–1.341	7.3x10^-1^	0.93	0.640–1.340	9.9x10^-1^	1.04	0.604–1.774	7.8x10^-1^	0.78	0.488–1.360	9.7x10^-1^	2.41	0.255–22.710	9.5x10^-1^	0.86	0.331–2.223
Possible insulin resistance	ADAMTS9	rs4607103	7.9x10^-1^	1.05	0.892–1.243	5.8x10^-1^	1.08	0.899–1.306	9.0x10^-1^	1.05	0.827–1.344	7.9x10^-1^	1.14	0.845–1.525	9.7x10^-1^	1.12	0.601–2.100	9.5x10^-1^	1.09	0.831–2.313
Obesity	FTO^5^	rs11642841	2.4x10^-1^	1.16	0.981–1.363	2.5x10^-1^	1.18	0.989–1.417	6.3x10^-1^	1.14	0.897–1.436	4.8x10^-1^	1.28	0.962–1.695	9.7x10^-1^	0.61	0.260–1.447	9.5x10^-1^	1.30	0.790–2.136
		rs8050136	8.1x10^-1^	1.04	0.881–1.218	7.5x10^-1^	1.04	0.864–1.240	9.2x10^-1^	0.96	0.751–1.219	9.7x10^-1^	1.02	0.776–1.345	9.7x10^-1^	1.25	0.649–2.390	9.5x10^-1^	1.11	0.688–1.790
		rs9939609	8.1x10^-1^	1.03	0.878–1.216	6.9x10^-1^	0.95	0.789–1.139	9.6x10^-1^	0.97	0.761–1.244	8.6x10^-1^	1.09	0.824–1.443	9.7x10^-1^	0.95	0.502–1.814	9.5x10^-1^	1.10	0.683–1.760
Circadian rhythm	CRY2	rs11605924	5.0x10^-1^	0.89	0.720–1.098	4.8x10^-1^	0.87	0.682–1.101	5.5x10^-1^	0.73	0.536–0.993	7.8x10^-1^	1.25	0.834–1.873	9.7x10^-1^	1.27	0.465–3.484	9.5x10^-1^	1.11	0.605–2.018

^1^ Q1 = Bedouin, Q2 = Persian/South Asian, Q3 = African, Admixed = Structure cut-off <0.65 in all sub-populations (k = 3).

^2^ Benjamini-Hochberg corrected p-value.

^3^ Estimated odds ratio (OR).

^4^ Lower and upper bounds of 95% confidence interval (CI) for odds ratio.

^5^ No BMI covariate used for FTO gene.

### Deep Sequencing of Qatari Genomes

One hundred Q1, Q2, Q3 Qatari genomes were sequenced to a median depth of 37x (minimum 30x) using Illumina reads on a HiSeq 2000 (Illumina Inc., San Diego, CA), as previously described [13]. Reads were mapped to the 1,000 Genomes Project version of the hg19/GRCh37 human reference genome using BWA 0.5.9 [14]. Sufficient paired-end 100 bp reads were generated in order to produce a median of 112 GB of sequence data passing filters and aligned to the hg19/GRCh37 human reference genome with a median insert size of 301 bp, where at least 85% of bases with quality score ≥30 (Q30) passed filtering steps and were aligned. Among non-N bases in the reference genome, at least 98% were covered by at least one base in all 100 genomes. Mapped reads were prepared for genotyping using the “Best practices for variant detection v3” GATK (http://www.broadinstitute.org/gatk/) pipeline, including removal of PCR-duplicate reads, realignment across known indels, and base quality score recalibration [15]. The number of exome bases covered at ≥1x and ≥10x was determined using SAMtools [16].

### Statistical Analysis

The PLINK software package (v1.07; http://pngu.mgh.harvard.edu/purcell/plink/) was used for statistical analyses [17]. First a pairwise similarity was calculated between all subjects using the 48 ancestry informative genotypes [8, 9], then a multi-dimensional scaling (MDS) algorithm was used to generate ethnic covariates for the case *vs* control logistic regression. To establish the set of MDS variables that achieved optimal separation between the sub-populations of Qataris we used the Akaike Information Criterion (AIC) as a means for model selection. Age, gender, BMI and T2D phenotypes were assigned as fixed effects and multi-dimensional scaling factors were sequentially added to the model. We found that the AIC scores converged once five or more multi-dimensional factors were introduced to the model.

The logistic regression analysis was performed using PLINK with and without BMI for each SNP individually under the additive genetic model. Other covariates included age, gender, and the first 5 MDS ethnic covariates as described above. SupportMix, a machine learning method for admixture analysis [12], was utilized to determine the population categorization (African, European or Asian) of Qatari haplotypes in regions flanking tag SNPs.

### Power Calculation

Genetic power calculations for 37 SNPs previously linked to T2D were conducted using the Genetic Power Calculator (GPC) [18]. The GPC analysis for case-control study of discrete traits takes as input the high risk allele frequency (based on TaqMan genotyping of Qataris), the disease prevalence (22% in Qatar), the genotype relative risk for heterozygotes [approximately equal to the odds ratio (OR)], the genotype relative risk for homozygotes (approximately the square of the OR), the D-prime linkage disequilibrium between the marker and risk variants (assumed to be 1.0), the marker allele frequency (risk allele frequency), number of cases, and the control:case ratio. The OR for the known T2D associated risk alleles ranged from 1.01 to 2.17 in other populations [19, 20], and prior studies of ethnic variance in OR suggests that the directionality is expected to be the same in Qataris [21, 22]. In the analysis, two scenarios were considered, one where the OR in Qatar is the same as in population of the reported SNP, and one where the OR in Qatar is equal to the highest reported OR among the 37 SNPs (2.71). In the first scenario, the n of cases needed for 80% higher was calculated and compared to our study, and in the second scenario the power given our sample size was calculated (S2 Table). Additionally, for each SNP, the minimum OR needed to detect an association with >80% power, given the sample size was calculated using a range of OR between 1.01 and 2.17, in 0.01 step intervals. The lowest OR with >80% power was reported (S2 Table). The minimum OR ranged from 1.18–1.44, with a mean of 1.25. The low end of this range (1.18), matched the mean OR of the reported SNPs (also 1.18), hence it was assumed that the OR could be 1.18 or higher in Qatar, as high as 2.17.

## Results

A total of 1,714 subjects were recruited from diabetes clinics and other departments in Hamad Medical Corporation in Doha, Qatar (S3 Table). All were self-reported third generation native Qatari over the age of thirty. Of these, 1,124 were classified as cases of T2D based on ADA criteria for fasting plasma glucose, oral glucose tolerance test or HbA1C level, without evidence of type 1 diabetes, MODY or MIDD as detailed in Methods. Forty percent (235/590) of control subjects had at least one first degree relative with TD2. Blood glucose and/or HbA1C data was available for 77% (455/590) of control subjects, providing information about their diabetes “at risk” status. Based on this, 53% (181/336) of control subjects were at risk with a HbA1C of 5.7–6.4%, and 25% (71/284) were at risk based on a random blood glucose level >5.6 mmol/L. One-hundred and sixty-six control subjects had both blood glucose and HbA1C levels measured, and of these, 29 (17%) were at risk by both criteria. The majority of the subjects were female both for cases with T2D (677/1,124; 60%) and controls (377/590; 64%; p = 0.14). T2D cases were significantly older with a mean age of 55 ± 10 yr *vs* 46 ± 9 yr in controls (p<10^−10^). BMI was significantly higher in T2D cases (34 ± 7 kg/m^2^) compared to controls (32 ± 7 kg/m^2^; p = 1.4x10^-6^). HbA1C levels were also significantly higher in T2D cases (8.3 ± 1.9) compared to controls (5.6 ± 0.4; p<10^−10^).

For all subjects, DNA was extracted from blood and Qatari subpopulation was determined by the use of 48 ancestry informative SNPs [8, 9]. Forty-five % of subjects were assigned as Q1 (Bedouin), 34% as Q2 (Persian, South Asian), and 8% as Q3 (African), leaving 13% classified as admixed (S1 Fig). The proportion of each subpopulation in T2D cases and controls was similar (p = 9.4x10^-1^).

**Fig 1:**
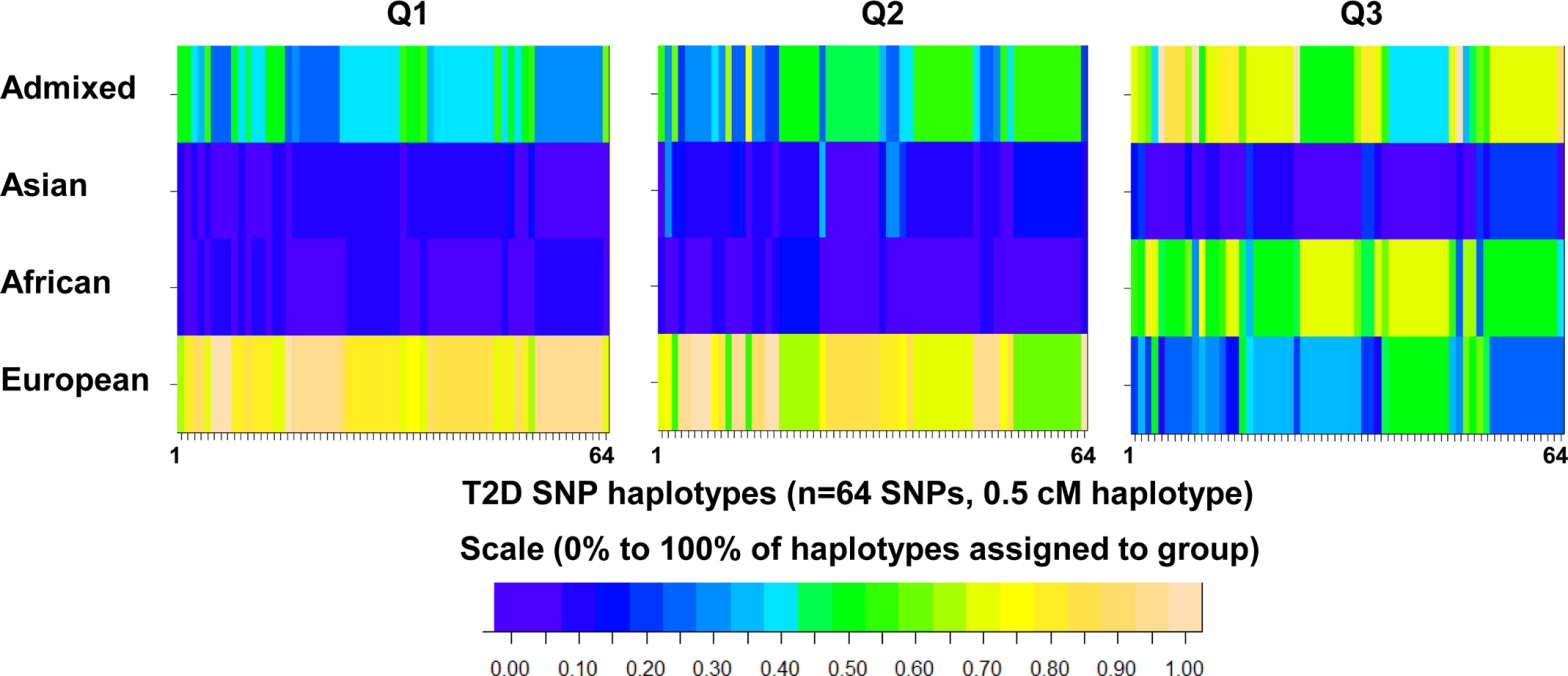
Comparison of Qatari haplotypes in the regions flanking known single nucleotide polymorphisms (SNPs) associated with type 2 diabetes (T2D) or nearby tag SNPs to the relevant haplotypes of European, Asian, African, or Admixed 1000 Genomes populations. Admixture deconvolution was used to determine if Qatari haplotypes flanking 64 ‘T2D SNPs’ matched the populations where these SNPs were discovered. Haplotypes were inferred for 100 deeply sequenced Qatari genomes (n = 60 Bedouin Q1, n = 20 Persian Q2, n = 20 African Q3), and divided into 2000 SNP (0.5 cM) intervals. For each interval, SupportMix [12] was used to infer the population with the most similar haplotype, using 1000 Genomes Project Phase 1 as a reference (Admixed = ASW, PUR, CLM, MXL; Asian = CHB, CHS, JPT; African = LWK, YRI; European = TSI, IBS, CEU, FIN, GBR). For each interval containing a T2D risk SNP (n = 64, S1 Table), the percentage of Qatari haplotypes assigned to European, Asian, African, or Admixed was determined. Shown is a heatmap of the results, where each column represents a SNP, and each row represents a 1000 Genomes group. Scaled from blue (0%) to tan (100%), colors represent the % of haplotypes for the SNP that are most similar to each Qatari population (Q1, Q2, or Q3).

We then chose 37 index SNPs known to be associated with risk for T2D based on GWAS studies performed in Europeans and South Asians [3, 6, 10] (Table 1). Of the 37 SNPs selected for genotyping in Qataris, the majority were associated with T2D in previous GWAS, with many of the susceptibility loci replicated in subsequent independent studies and meta-analyses. As the genetic risks for T2D in Qataris are unknown, SNPs were chosen from GWAS performed in both European and Asian populations. An additional 27 tag SNPs with high minor allele frequency adjacent to these loci were also chosen to obtain more information on Qatari haplotypes (S1 Table). Qatari haplotypes in the region of these SNPs were compared to the haplotypes of other populations to ensure that these SNPs were potentially relevant to Qatari’s prior to genotyping a large number of subjects. Using the 1000 Genomes Project Phase 1 as a reference, haplotype blocks in the regions flanking these 64 SNPs in Europeans, Africans and Asians were compared to Qatari subpopulation haplotype blocks. This analysis revealed that the T2D haplotypes of Qataris from Q1 and Q2 subpopulations were similar to European T2D haplotypes in (Fig 1). In contrast, the majority of T2D haplotypes in the Q3 subpopulation were categorized as African.

**Fig 2:**
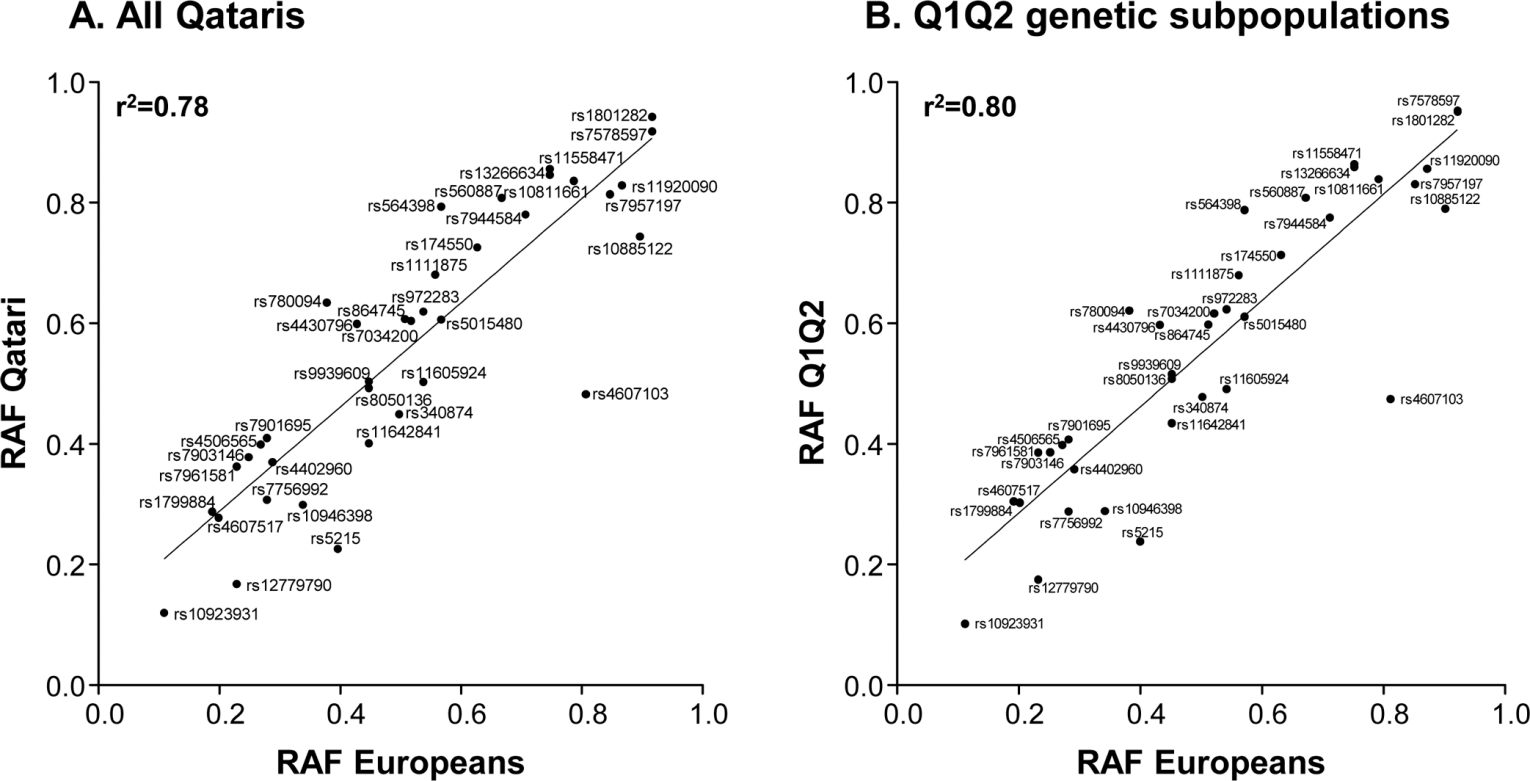
Population risk allele frequencies. Population risk allele frequencies (RAF) in **A.** all Qataris, and **B.** Q1 and Q2 genetic subpopulations combined, compared to European RAF (obtained from Hapmap [23]) for 37 SNPs previously associated with T2D, with the straight line indicating the regression line of best fit of the data.

**Fig 3:**
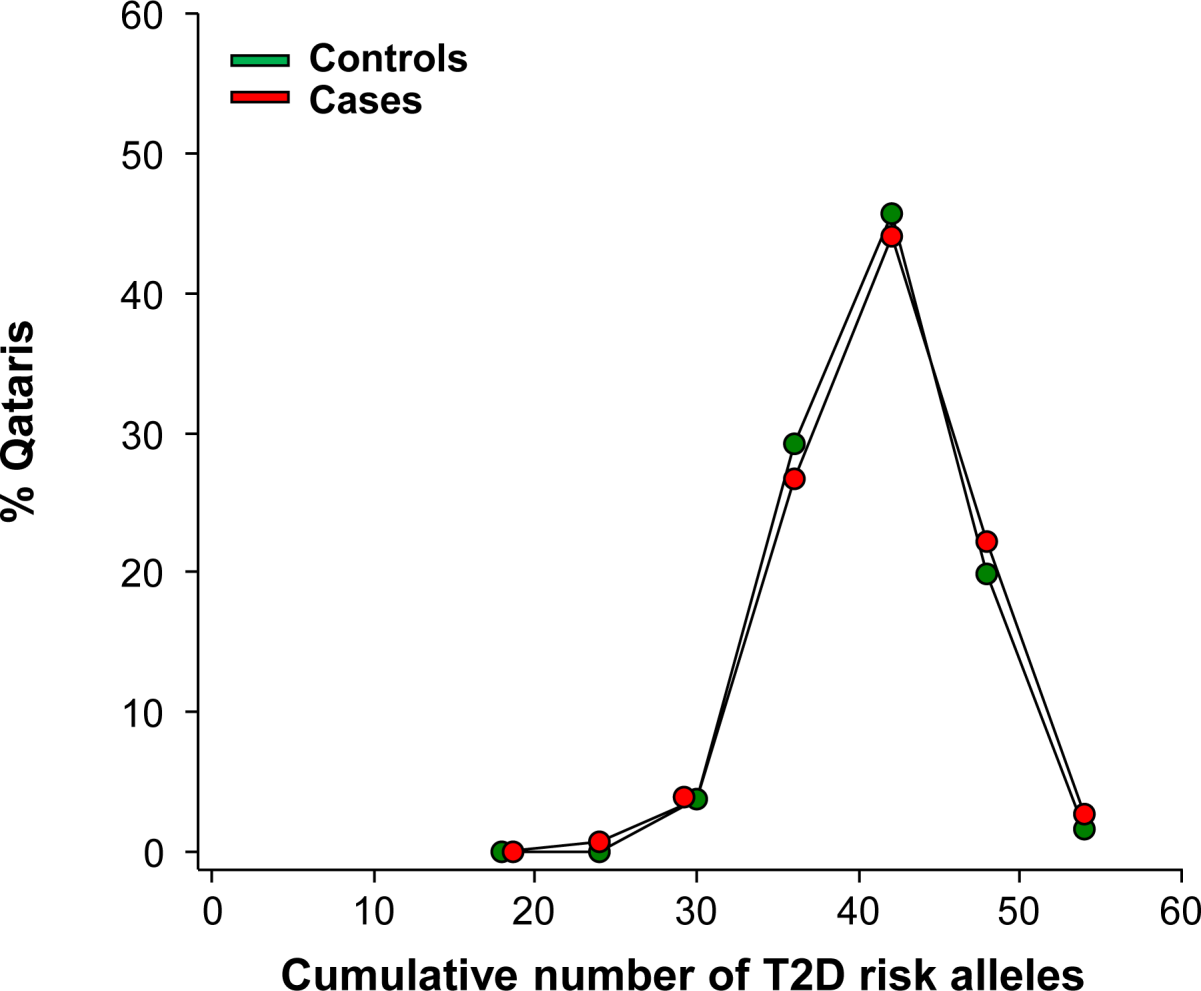
Cumulative distribution of 37 Type 2 Diabetes (T2D) risk allele counts. Cumulative distribution of 37 T2D risk allele counts in Qataris (n = 952) cases (red, n = 626), and controls (green, n = 326). Two-tailed, unequal variance t-test, p = 0.26.

Given the high prevalence of T2D in Qatar, and the similarities in T2D haplotypes between Qataris and Europeans, we genotyped 1,714 subjects (1,124 cases and 590 controls) for these established T2D risk alleles. Following quality control analysis, the risk allele frequency (RAF) of each SNP was determined. Then, the RAF of 37 T2D associated SNPs in Qataris were compared to those of Europeans (obtained from HapMap; Fig 2A) [23]. There was good overall correlation between Qatari and European RAF (Pearson correlation coefficient 0.89, r^2^ = 0.78, p<10^−4^), although the RAF was higher in Qataris than Europeans for 70% of SNPs (26/37). Of the 37 SNPs with known risk alleles, the SNP with the highest RAF in all Qataris was rs1801282 in PPARG (peroxisome proliferator-activated receptor gamma) with a RAF of 0.94. SNP rs7903146 in transcription factor 7-like 2 (TCF7L2), the SNP most strongly associated with T2D in other populations [24], had a RAF in all Qataris of 0.37, higher than the RAF observed in Europeans (0.25). Since the haplotype analysis revealed that the Q1 and Q2 genetic subpopulations were most similar to Europeans in the region of T2D associated SNPs, the RAF frequencies in these subpopulations were combined and compared to Europeans. The combined Q1 and Q2 RAF were in closer agreement with European RAF for T2D associated SNPs (Pearson correlation coefficient 0.89, r^2^ = 0.8, p<10^−4^, Fig 2B) than were all Qataris. When the distribution of risk alleles in 952 Qatari cases and controls (of all genetic subpopulations) who had undergone genotyping for all 37 T2D associated SNPs was examined, 47% of all subjects had ≥40 T2D associated alleles (Fig 3). There was no significant difference between the number of risk alleles in cases compared to controls (p>0.2).

Assuming the OR in Qatar is equal to the highest reported OR (2.17) among the 37 SNPs with known risk alleles, given our sample size, there was >80% power to detect an association for a given SNP, with a type I error rate of 0.05 (S2 Table). The difference in allele frequencies between T2D cases and controls was then determined (Table 1). Age, BMI and gender were introduced as covariates in the analysis since they differed significantly between cases and controls (S3 Table), as well as the first 5 MDS ethnic covariates. As the Q1 and Q2 genetic subpopulations were most similar to Europeans, in addition to analyzing each subpopulation individually, these subpopulations were also combined and analyzed. However, despite the high RAF observed in Qataris, after Benjamini-Hochberg (BH) adjustment for multiple testing, only two SNPs, both in transcription factor 7-like 2 (TCF7L2) achieved statistical significance in the whole study population, rs7903146 (p = 0.028) and rs4506565 (p = 0.036). When T2D subjects and control subjects were assigned to the Qatari subpopulations, one of these SNPs (rs4506565) was also significant in the admixed group (p = 0.045; Table 1).

Twenty-seven tag SNPs adjacent to the known T2D associated SNPs were also genotyped in all Qataris, and in a subgroup of subjects, genotyping was performed for the additional 16 SNPs in 15 genes associated with T2D in Europeans and South Asians (S1 Table). For these SNPs, a similar analysis was conducted comparing allele frequencies between cases and controls for all Qataris and individual genetic subpopulations, including Q1 and Q2 combined. Significance was not observed in any additional SNPs among the Qatari population as a whole, although the SNP rs4074718 in TCF7L2 (p<0.05) was significant in the subpopulation defined as admixed (S1 Table). For all other SNPs confidence intervals for ORs gave no indication that these SNPs contribute to diabetes risk in Qataris (S1 Table).

## Discussion

Qatar has one of the highest incidences of T2D in the world [7]. Despite sharing similar haplotypes and risk allele frequency (RAF) with Europeans, the T2D SNPs commonly associated with T2D in Europeans and Asians do not help explain the very high prevalence of T2D in Qataris. This observation emphasizes the importance of population-specific genetic variation in the pathogenesis of common disorders such as T2D.

### T2D Risk Alleles in Europeans and Asians

To date, GWAS have identified over 90 loci associated with T2D [3–6, 10], helping to understand the pathogenesis of T2D, identifying novel metabolic pathways and potential targets for therapy [5]. The SNP that is most strongly associated with T2D is rs7903146 in TCF7L2, a transcription factor related to insulin and proglucagon genes [3, 24–26]. The association between TCF7L2 and T2D has been reproduced in various ethnic groups [24], including the Qataris in this study. However, despite the robust association between TCF7L2 and T2D, the causal variant has not yet been identified [27]. Other loci strongly associated with T2D include hematopoietically expressed homeobox (HHEX), peroxisome proliferator-activated receptor gamma (PPARG), solute carrier family 30 (zinc transporter) member 8 (SCL30A8), CDK5 regulatory subunit associated protein 1-like 1 (CDKAL1), and fat mass and obesity-associated protein (FTO) [3–6, 10]. Most of these GWAS have been carried out in European and Asian populations, and given the significant differences in genetic architecture in different populations [28, 29], the loci identified may not be transferable to other populations.

### T2D Risk Alleles in Middle Eastern Populations

There have been no prior GWAS of T2D risk alleles in Middle Eastern populations, but there have been several candidate genes studies, albeit with inconsistent results. While it has been reported that many of the known European risk alleles are associated with T2D in Lebanese Arabs [30], the Saudi Arabian population [31], and in Tunisians and Moroccans with cumulative effects [32], other studies have not demonstrated an association between the European T2D risk SNPs in these populations. For example, though a link between SNPs in TCF7L2 and T2D has been reported in Moroccans [32], only a marginal association has been found in Arabs [33], and several European SNPs were not associated with T2D in Tunisians [34]. Similarly, only 2 of 23 loci associated with BMI in other populations have been linked to obesity in Qataris [35].

Consistent with these observations, despite demonstrating that T2D haplotypes of Q1 and Q2 Qataris are more similar to European than African and Asian haplotypes, we found that only two SNPs in TCF7L2, the susceptibility gene most strongly associated with T2D in Europeans, were associated with T2D in all Qataris. This may be explained by variations in patterns of linkage disequilibrium (LD) between SNPs and susceptibility loci, whereby tag SNPs may be transferable between populations as demonstrated by the similarity between Q1 and Q2 Qatari and European T2D haplotypes, while the haplotype blocks may still vary sufficiently so that the causal variants are not in LD with tag SNPs [36, 37].

Diabetes results from complex interactions between genetic, environmental and behavioral factors [1, 2]. Most of the T2D susceptibility genes identified by GWAS to date relate to beta-cell dysfunction rather than insulin resistance [3–6, 10]. In contrast to many of the subjects included in prior GWAS studies, Qataris have been subject to rapidly changing environmental factors including Westernization of diet, and an increasingly sedentary lifestyle, with consequent high rates of obesity [38, 39]. There may be population specific environmental factors associated with disease risk or protection [40], and perhaps insulin resistance pathways may play a greater role in the development of T2D in these subjects than other populations.

### Population Risk Allele Frequencies

There was significant correlation between the observed RAF in Qataris and Europeans, consistent with previous reports of a directionally similar association to T2D between populations [21, 41, 42]. Despite this finding, only 2 of 37 known T2D risk alleles were associated with T2D in Qataris, suggesting they are not responsible for the condition in this population. This finding highlights the importance of population-specific variation in the pathogenesis of common condition such as T2D, and the need for genetic studies in diverse populations.

GWAS identifies common variants with a low OR [43], and it is believed that multiple SNPs in an individual are required for the pathogenesis of complex conditions such as T2D [44]. The risk allele frequency in Qataris for the 37 SNPs associated with T2D ranged from 0.12 to 0.94 (mean 0.55), indicating that the risk variants were at a high frequency within both cases and controls. In fact, 47% of subjects carried ≥40 risk alleles. Additionally, the risk variants were individually associated with a low OR (maximum OR 1.36, TCF7L2 rs7903146), with each SNP conferring only a small risk of T2D. As a large number of the subjects carry multiple risk alleles without developing T2D, this low penetrance suggests that each variant had only a mild effect, and that other factors such as diet and lifestyle are required for development of T2D.

## Supporting Information

S1 Fig Scatter plot of multidimensional scaling (MDS) illustrating subject distribution by Qatari genetic subpopulation. (PDF)

S1 References References Used in Supplemental Data. (PDF)

S1 Table Type 2 Diabetes Risk Allele SNPs. (PDF)

S2 Table Statistical Power to Detect Associations with Type 2 Diabetes for Known Risk Alleles (with references). (PDF)

S3 Table Demographics of Type 2 Diabetic Cases and Controls. (PDF)
