# Correction: Type 2 Diabetes Risk Allele Loci in the Qatari Population

**DOI:** 10.1371/journal.pone.0161910

**Published:** 2016-08-23

**Authors:** Sarah L. O’Beirne, Jacqueline Salit, Juan L. Rodriguez-Flores, Michelle R. Staudt, Charbel Abi Khalil, Khalid A. Fakhro, Amal Robay, Monica D. Ramstetter, Iman K. Al-Azwani, Joel A. Malek, Mahmoud Zirie, Amin Jayyousi, Ramin Badii, Ajayeb Al-Nabet Al-Marri, Maria J. Chiuchiolo, Alya Al-Shakaki, Omar Chidiac, Maey Gharbiah, Abdulbari Bener, Dora Stadler, Neil R. Hackett, Jason G. Mezey, Ronald G. Crystal

The first five authors—Sarah L. O’Beirne, Jacqueline Salit, Juan L. Rodriguez-Flores, Michelle R. Staudt, Charbel Abi Khalil—should be noted as contributing equally to this work. The fifth author, Charbel Abi Khalil, is incorrectly designated as the corresponding author. The correct corresponding author is Ronald G. Crystal. The corresponding author’s email address is: geneticmedicine@med.cornell.edu
