# Supplementary material for: Type 2 Diabetes Risk Allele Loci in the Qatari Population
Source: PLoS One. 2016 Jul 6;11(7):e0156834. doi: 10.1371/journal.pone.0156834 (PMC4934876; doi:10.1371/journal.pone.0156834)
Supplement: S1 Fig — (PDF) [file pone.0156834.s001.pdf]

S1 Fig

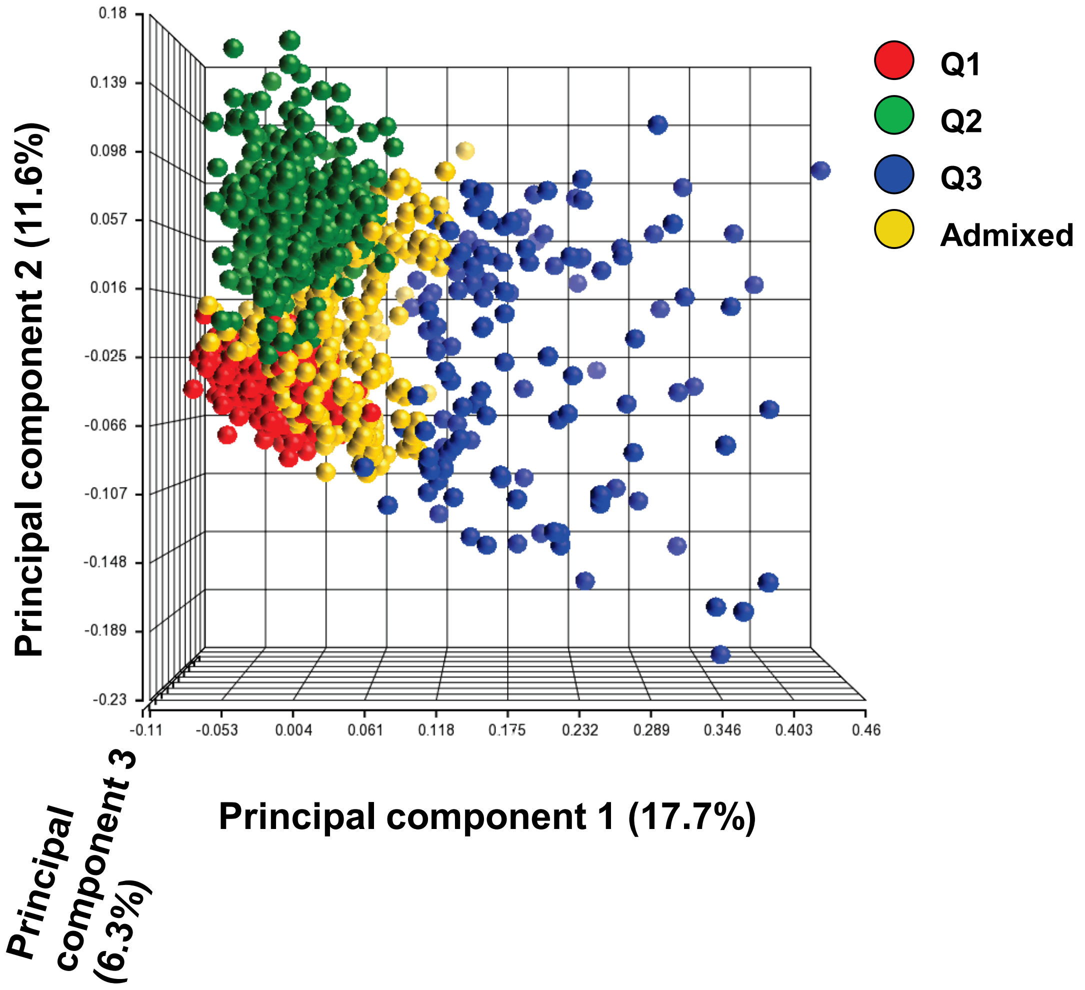

S1 Fig. Scatter plot of multidimensional scaling (MDS) illustrating subject distribution by Qatari genetic subpopulation. Shown is MDS on pair-wise similarities between 1,714 unrelated Qataris subjects with K=3 populations, generated using Structure v2.3. The plot illustrates Qatari genetic subpopulation structure, where a proportion of ≥ 65% was required to be assigned to Q1 (Bedouin), Q2 (Persian-South Asian), or Q3 (African) genetic subgroups; <65% assigned to “Admixed” subgroup. Individuals are color-coded by genetic subpopulation (Q1 = red, Q2 = green, Q3 = blue, Admixed = yellow).
