# Supplementary material for: Type 2 Diabetes Risk Allele Loci in the Qatari Population
Source: PLoS One. 2016 Jul 6;11(7):e0156834. doi: 10.1371/journal.pone.0156834 (PMC4934876; doi:10.1371/journal.pone.0156834)
Supplement: S1 References — (PDF) [file pone.0156834.s002.pdf]

SI References

1. Zeggini E, Scott LJ, Saxena R, Voight BF, Marchini JL, Hu T, et al. Meta-analysis of genome-wide association data and large-scale replication identifies additional susceptibility loci for type 2 diabetes. Nat Genet. 2008;40: 638-645

2. Zeggini E, McCarthy MI. Identifying susceptibility variants for type 2 diabetes. Methods Mol Biol. 2007;376: 235-250

3. Steinthorsdottir V, Thorleifsson G, Reynisdottir I, Benediktsson R, Jonsdottir T, Walters GB, et al. A variant in CDKAL1 influences insulin response and risk of type 2 diabetes. Nat Genet. 2007;39: 770-775

4. Saxena R, Gianniny L, Burtt NP, Lyssenko V, Giuducci C, Sjogren M, et al. Common single nucleotide polymorphisms in TCF7L2 are reproducibly associated with type 2 diabetes and reduce the insulin response to glucose in nondiabetic individuals. Diabetes. 2006;55: 2890-2895

5. Dupuis J, Langenberg C, Prokopenko I, Saxena R, Soranzo N, Jackson AU, et al. New genetic loci implicated in fasting glucose homeostasis and their impact on type 2 diabetes risk. Nat Genet. 2010;42: 105-116

6. Takeuchi F, Serizawa M, Yamamoto K, Fujisawa T, Nakashima E, Ohnaka K, et al. Confirmation of multiple risk Loci and genetic impacts by a genome-wide association study of type 2 diabetes in the Japanese population. Diabetes. 2009;58: 1690-1699

7. Voight BF, Scott LJ, Steinthorsdottir V, Morris AP, Dina C, Welch RP, et al. Twelve type 2 diabetes susceptibility loci identified through large-scale association analysis. Nat Genet. 2010;42: 579-589

8. Vaxillaire M, Veslot J, Dina C, Proenca C, Cauchi S, Charpentier G, et al. Impact of common type 2 diabetes risk polymorphisms in the DESIR prospective study. Diabetes. 2008;57: 244-254

9. Liu C, Li H, Qi L, Loos RJ, Qi Q, Lu L, et al. Variants in GLIS3 and CRY2 are associated with type 2 diabetes and impaired fasting glucose in Chinese Hans. PLoS One. 2011;6: e21464

10. Xu J, Wang J, Chen B. SLC30A8 (ZnT8) variations and type 2 diabetes in the Chinese Han population. Genetics and molecular research: GMR. 2012;11: 1592-1598

11. Sladek R, Rocheleau G, Rung J, Dina C, Shen L, Serre D, et al. A genome-wide association study identifies novel risk loci for type 2 diabetes. Nature. 2007;445: 881-885

12. Wellcome Trust Case Control C. Genome-wide association study of 14,000 cases of seven common diseases and 3,000 shared controls. Nature. 2007;447: 661-678

13. Grant SF, Thorleifsson G, Reynisdottir I, Benediktsson R, Manolescu A, Sainz J, et al. Variant of transcription factor 7-like 2 (TCF7L2) gene confers risk of type 2 diabetes. Nat Genet. 2006;38: 320-323

14. Qi Q, Wu Y, Li H, Loos RJ, Hu FB, Sun L, et al. Association of GCKR rs780094, alone or in combination with GCK rs1799884, with type 2 diabetes and related traits in a Han Chinese population. Diabetologia. 2009;52: 834-843
