# Supplementary material for: Type 2 Diabetes Risk Allele Loci in the Qatari Population
Source: PLoS One. 2016 Jul 6;11(7):e0156834. doi: 10.1371/journal.pone.0156834 (PMC4934876; doi:10.1371/journal.pone.0156834)
Supplement: S1 Table — (PDF) [file pone.0156834.s003.pdf]

S1 Table. Type 2 Diabetes Risk Allele SNPs

			All Qatari			Q1 +Q2[1]			Q1[1]			Q2[1]			Q3[1]			Admixed[1]		
**Category**	**Gene**	**SNP**	**p value[2]**	**OR[3]**	**CI[4]**	**p value[2]**	**OR[3]**	**CI[4]**	**p value[2]**	**OR[3]**	**CI[4]**	**p value[2]**	**OR[3]**	**CI[4]**	**p value[2]**	**OR[3]**	**CI[4]**	**p value[2]**	**OR[3]**	**CI[4]**
Beta-cell dysfunction	CAMK1D/CDC123	rs12779790	5.0x10^{-1}	1.13	0.909-1.401	2.5x10^{-1}	1.26	0.992-1.603	5.5x10^{-1}	1.33	0.972-1.812	8.0x10^{-1}	1.16	0.792-1.685	9.7x10^{-1}	0.45	0.174-1.176	9.5x10^{-1}	0.85	0.442-1.631
	CDKAL1	rs10946398	4.3x10^{-1}	1.13	0.931-1.364	5.5x10^{-1}	1.12	0.905-1.394	9.9x10^{-1}	1.01	0.764-1.340	4.8x10^{-1}	1.32	0.935-1.859	9.7x10^{-1}	1.50	0.713-3.158	9.5x10^{-1}	1.23	0.704-2.158
		rs7756992	8.0x10^{-1}	1.06	0.840-1.327	5.8x10^{-1}	1.13	0.856-1.469	9.9x10^{-1}	1.01	0.718-1.420	4.8x10^{-1}	1.45	0.919-2.278	9.7x10^{-1}	1.14	0.485-2.672	9.5x10^{-1}	0.71	0.355-1.411
	CDKN2A-B	rs545226	1.8x10^{-1}	0.84	0.709-0.989	1.8x10^{-1}	0.79	0.657-0.949	2.6x10^{-1}	0.69	0.535-0.889	8.8x10^{-1}	0.93	0.708-1.216	9.7x10^{-1}	1.40	0.632-3.093	9.5x10^{-1}	0.79	0.481-1.284
		rs564398	7.6x10^{-1}	0.94	0.772-1.134	5.6x10^{-1}	0.90	0.727-1.107	6.4x10^{-1}	0.87	0.651-1.151	9.1x10^{-1}	0.94	0.689-1.293	9.7x10^{-1}	1.33	0.418-4.224	9.5x10^{-1}	1.08	0.599-1.961
		rs12376000	1.4x10^{-1}	0.64	0.445-0.923	1.8x10^{-1}	0.56	0.375-0.842	5.0x10^{-1}	0.57	0.343-0.942	4.8x10^{-1}	0.54	0.271-1.074	9.7x10^{-1}	1.62	0.118-22.240	9.5x10^{-1}	1.09	0.394-3.001
		rs944801	5.7x10^{-1}	0.91	0.746-1.105	4.8x10^{-1}	0.88	0.709-1.091	5.5x10^{-1}	0.83	0.619-1.113	9.1x10^{-1}	0.94	0.686-1.304	9.7x10^{-1}	0.96	0.305-3.008	9.5x10^{-1}	1.02	0.564-1.858
		rs10757269	1.5x10^{-1}	0.81	0.670-0.968	1.8x10^{-1}	0.80	0.651-0.976	5.5x10^{-1}	0.81	0.615-1.079	4.8x10^{-1}	0.78	0.583-1.049	9.7x10^{-1}	0.56	0.220-1.404	9.5x10^{-1}	1.04	0.595-1.811
		rs7341786	1.9x10^{-1}	0.84	0.697-1.006	2.8x10^{-1}	0.84	0.686-1.025	5.5x10^{-1}	0.82	0.624-1.073	7.8x10^{-1}	0.87	0.647-1.175	9.7x10^{-1}	0.91	0.336-2.480	9.5x10^{-1}	0.82	0.468-1.447
		rs10811661	1.9x10^{-1}	0.81	0.651-1.002	3.3x10^{-1}	0.82	0.645-1.039	5.5x10^{-1}	0.82	0.595-1.125	7.8x10^{-1}	0.82	0.567-1.176	7.4x10^{-1}	0.18	0.049-0.683	9.5x10^{-1}	0.98	0.531-1.804
		rs10757284	7.9x10^{-1}	1.04	0.880-1.238	5.6x10^{-1}	1.10	0.908-1.339	5.5x10^{-1}	1.18	0.906-1.537	9.7x10^{-1}	1.01	0.755-1.345	1.0x10^{0}	1.00	0.528-1.893	9.5x10^{-1}	0.83	0.499-1.381
	G6PC2	rs560887	9.7x10^{-1}	1.01	0.753-1.305	6.7x10^{-1}	0.90	0.819-1.515	8.4x10^{-1}	0.89	0.767-1.640	9.1x10^{-1}	1.11	0.510-1.596	9.7x10^{-1}	0.79	0.358-4.474	9.5x10^{-1}	1.67	0.246-1.440
	HHEX/IDE/KIF11	rs1111875	1.8x10^{-1}	0.82	0.685-0.979	1.8x10^{-1}	0.79	0.649-0.968	9.0x10^{-1}	0.94	0.719-1.232	2.1x10^{-1}	0.63	0.465-0.859	9.7x10^{-1}	1.29	0.614-2.725	9.5x10^{-1}	0.75	0.443-1.269
	HNF1A	rs7957197	2.7x10^{-1}	0.80	0.611-1.058	4.0x10^{-1}	0.81	0.589-1.101	6.9x10^{-1}	0.83	0.550-1.240	8.6x10^{-1}	0.85	0.505-1.427	9.7x10^{-1}	1.72	0.488-6.040	9.5x10^{-1}	0.67	0.294-1.516
	HNF1B	rs4430796	1.8x10^{-1}	0.80	0.649-0.991	1.8x10^{-1}	0.75	0.595-0.953	5.0x10^{-1}	0.71	0.521-0.962	7.8x10^{-1}	0.83	0.567-1.223	9.7x10^{-1}	1.21	0.422-3.445	9.5x10^{-1}	0.84	0.430-1.639
	JAZF1	rs864745	1.4x10^{-1}	0.81	0.686-0.957	2.4x10^{-1}	0.83	0.691-0.999	9.6x10^{-1}	0.96	0.755-1.230	2.1x10^{-1}	0.68	0.514-0.909	9.7x10^{-1}	0.69	0.308-1.546	9.5x10^{-1}	0.92	0.560-1.508
	KCNJ11	rs5215	8.1x10^{-1}	1.04	0.853-1.258	7.5x10^{-1}	1.04	0.841-1.293	9.0x10^{-1}	1.07	0.794-1.447	9.4x10^{-1}	1.04	0.762-1.431	9.7x10^{-1}	0.60	0.173-2.061	9.5x10^{-1}	1.05	0.615-1.779
	MADD	rs7944584	8.0x10^{-1}	0.94	0.723-1.224	7.5x10^{-1}	1.06	0.792-1.421	5.5x10^{-1}	1.37	0.934-2.008	7.8x10^{-1}	0.75	0.463-1.223	9.7x10^{-1}	0.23	0.045-1.220	5.9x10^{-1}	0.52	0.249-1.089
	NOTCH2	rs10923931	7.9x10^{-1}	1.08	0.825-1.399	5.8x10^{-1}	1.16	0.846-1.597	9.9x10^{-1}	1.01	0.646-1.563	6.9x10^{-1}	1.35	0.847-2.143	9.7x10^{-1}	0.96	0.453-2.050	9.5x10^{-1}	1.06	0.506-2.222
	THADA	rs7578597	7.6x10^{-1}	0.90	0.667-1.207	5.6x10^{-1}	0.83	0.570-1.197	9.0x10^{-1}	0.87	0.465-1.620	8.0x10^{-1}	0.84	0.527-1.325	9.7x10^{-1}	1.50	0.693-3.244	9.5x10^{-1}	0.95	0.462-1.952
Reduced insulin secretion	ADRA2A	rs10885122	2.4x10^{-1}	0.82	0.642-1.036	4.0x10^{-1}	0.82	0.620-1.089	5.5x10^{-1}	0.73	0.514-1.042	1.0x10^{0}	1.00	0.609-1.640	9.7x10^{-1}	0.96	0.415-2.232	9.5x10^{-1}	0.68	0.345-1.327
	FADS1	rs174550	1.9x10^{-1}	1.26	0.998-1.585	1.8x10^{-1}	1.33	1.026-1.710	5.5x10^{-1}	1.28	0.935-1.757	4.8x10^{-1}	1.44	0.918-2.262	9.7x10^{-1}	0.94	0.214-4.097	9.5x10^{-1}	1.11	0.558-2.220
	GCK	rs1799884	9.4x10^{-1}	1.02	0.802-1.287	7.1x10^{-1}	0.93	0.715-1.214	9.6x10^{-1}	1.04	0.742-1.444	6.9x10^{-1}	0.74	0.461-1.182	9.7x10^{-1}	1.06	0.358-3.150	9.5x10^{-1}	1.35	0.642-2.840
		rs4607517	9.8x10^{-1}	1.00	0.786-1.265	6.4x10^{-1}	0.91	0.695-1,179	9.9x10^{-1}	1.00	0.720-1.395	6.8x10^{-1}	0.72	0.450-1.151	9.7x10^{-1}	1.61	0.434-5.992	9.5x10^{-1}	1.32	0.616-2.808
	GLIS3	rs7034200	2.7x10^{-1}	0.83	0.668-1.042	5.8x10^{-1}	0.89	0.689-1.141	5.5x10^{-1}	0.81	0.585-1.116	9.0x10^{-1}	1.11	0.726-1.697	9.7x10^{-1}	0.59	0.209-1.691	6.3x10^{-1}	0.58	0.303-1.107

S1 Table. Type 2 Diabetes Risk Allele SNPs (cont., page 2)

			All Qatari			Q1 +Q2[1]			Q1[1]			Q2[1]			Q3[1]			Admixed[1]		
**Category**	**Gene**	**SNP**	**p value[2]**	**OR[3]**	**CI[4]**	**p value[2]**	**OR[3]**	**CI[4]**	**p value[2]**	**OR[3]**	**CI[4]**	**p value[2]**	**OR[3]**	**CI[4]**	**p value[2]**	**OR[3]**	**CI[4]**	**p value[2]**	**OR[3]**	**CI[4]**
	LGR5/TSPAN8	rs7961581	6.5×10^{-1}	1.07	0.913-1.258	5.8×10^{-1}	1.09	0.909-1.296	5.5×10^{-1}	1.19	0.943-1.507	9.7×10^{-1}	0.97	0.740-1.283	9.7×10^{-1}	1.33	0.614-2.876	9.5×10^{-1}	1.02	0.633-1.632
	PROX1	rs340874	8.1×10^{-1}	1.04	0.843-1.281	5.8×10^{-1}	1.11	0.877-1.414	5.5×10^{-1}	1.23	0.901-1.669	9.0×10^{-1}	1.11	0.736-1.665	9.7×10^{-1}	0.71	0.233-2.164	9.5×10^{-1}	0.82	0.470-1.416
	SLC2A2 (GLUT2)	rs11920090	3.3×10^{-1}	1.24	0.922-1.657	3.5×10^{-1}	1.31	0.933-1.829	5.5×10^{-1}	1.40	0.909-2.165	9.1×10^{-1}	1.11	0.651-1.891	9.7×10^{-1}	2.41	0.719-8.059	9.3×10^{-1}	0.54	0.220-1.348
	SLC30A8	rs7815190	5.1×10^{-1}	1.12	0.908-1.386	5.9×10^{-1}	1.11	0.868-1.419	7.6×10^{-1}	1.14	0.823-1.571	9.4×10^{-1}	1.05	0.716-1.553	9.7×10^{-1}	1.59	0.707-3.569	9.5×10^{-1}	1.22	0.678-2.191
		rs4876703	1.9×10^{-1}	0.83	0.695-1.000	2.2×10^{-1}	0.80	0.651-0.989	5.5×10^{-1}	0.82	0.615-1.093	4.8×10^{-1}	0.77	0.564-1.044	9.7×10^{-1}	1.06	0.527-2.112	9.5×10^{-1}	0.98	0.602-1.600
		rs13281296	6.5×10^{-1}	0.91	0.736-1.129	5.8×10^{-1}	0.89	0.697-1.142	5.5×10^{-1}	0.83	0.629-1.104	8.0×10^{-1}	0.85	0.589-1.234	9.7×10^{-1}	0.57	0.262-1.226	9.5×10^{-1}	1.29	0.762-2.193
		rs2466296	7.9×10^{-1}	1.06	0.891-1.254	9.9×10^{-1}	1.00	0.821-1.211	9.6×10^{-1}	0.97	0.752-1.257	9.7×10^{-1}	1.03	0.760-1.382	9.7×10^{-1}	0.54	0.274-1.076	9.5×10^{-1}	1.04	0.649-1.679
		rs13266634	9.6×10^{-1}	1.02	0.738-1.396	6.9×10^{-1}	1.11	0.769-1.614	9.6×10^{-1}	1.07	0.590-1.942	8.1×10^{-1}	1.20	0.737-1.936	9.7×10^{-1}	0.21	0.044-1.005	9.5×10^{-1}	1.06	0.488-2.298
		rs11558471	2.7×10^{-1}	0.78	0.581-1.058	6.8×10^{-1}	0.89	0.637-1.252	9.9×10^{-1}	1.02	0.643-1.632	8.0×10^{-1}	0.80	0.470-1.367	9.7×10^{-1}	0.50	0.133-1.902	5.9×10^{-1}	0.43	0.171-1.096
		rs1578978	2.4×10^{-1}	1.16	0.978-1.368	3.3×10^{-1}	1.17	0.968-1.412	5.5×10^{-1}	1.17	0.907-1.507	7.8×10^{-1}	1.15	0.871-1.536	9.7×10^{-1}	0.69	0.308-1.530	8.4×10^{-1}	1.41	0.876-2.266
	TCF7L2	rs6585194	7.9×10^{-1}	0.95	0.786-1.142	6.0×10^{-1}	0.92	0.739-1.136	8.6×10^{-1}	0.92	0.684-1.236	8.6×10^{-1}	0.90	0.659-1.246	9.7×10^{-1}	0.64	0.313-1.314	9.5×10^{-1}	1.18	0.708-1.962
		rs7901275	1.4×10^{-1}	0.80	0.677-0.953	2.5×10^{-1}	0.84	0.691-1.013	5.5×10^{-1}	0.81	0.626-1.053	7.8×10^{-1}	0.85	0.641-1.138	9.7×10^{-1}	0.95	0.481-1.894	8.4×10^{-1}	0.69	0.409-1.149
		rs6585196	1.1×10^{-1}	0.78	0.650-0.926	1.8×10^{-1}	0.78	0.642-0.953	5.5×10^{-1}	0.85	0.654-1.108	2.7×10^{-1}	0.69	0.510-0.933	9.7×10^{-1}	0.70	0.348-1.409	9.5×10^{-1}	0.76	0.440-1.302
		rs4074718	2.4×10^{-1}	0.86	0.734-1.019	6.9×10^{-1}	0.95	0.786-1.137	9.6×10^{-1}	1.03	0.808-1.316	8.7×10^{-1}	0.92	0.693-1.225	9.7×10^{-1}	1.08	0.541-2.166	**4.3×10^{-2}**	0.43	0.262-0.697
		rs7901695	1.8×10^{-1}	1.20	1.016-1.414	3.6×10^{-1}	1.15	0.958-1.389	7.6×10^{-1}	1.11	0.866-1.412	6.8×10^{-1}	1.22	0.908-1.620	9.7×10^{-1}	0.95	0.473-1.916	1.2×10^{-1}	1.90	1.155-3.131
		rs4506565	**3.7×10^{-2}**	1.33	1.121-1.586	1.8×10^{-1}	1.24	1.023-1.507	5.5×10^{-1}	1.18	0.916-1.515	4.8×10^{-1}	1.34	0.986-1.820	9.7×10^{-1}	1.35	0.637-2.875	**4.5×10^{-2}**	2.43	1.409-4.195
		rs7903146	**2.9×10^{-2}**	1.36	1.146-1.618	1.8×10^{-1}	1.28	1.057-1.551	5.5×10^{-1}	1.20	0.932-1.537	3.4×10^{-1}	1.40	1.034-1.890	9.7×10^{-1}	1.18	0.503-2.779	7.4×10^{-2}	2.17	1.291-3.649
		rs6585198	1.4×10^{-1}	0.81	0.683-0.962	4.0×10^{-1}	1.15	0.947-1.388	6.4×10^{-1}	1.14	0.886-1.460	7.8×10^{-1}	0.87	0.644-1.175	9.7×10^{-1}	0.87	0.435-1.747	9.3×10^{-2}	0.48	0.288-0.810
		rs7924080	1.8×10^{-1}	0.84	0.709-0.987	3.8×10^{-1}	0.87	0.725-1.047	6.3×10^{-1}	1.14	0.893-1.451	7.8×10^{-1}	0.87	0.656-1.160	9.7×10^{-1}	1.05	0.518-2.144	1.2×10^{-1}	0.52	0.317-0.862
		rs7900150	2.4×10^{-1}	0.87	0.736-1.020	4.8×10^{-1}	0.90	0.746-1.074	7.8×10^{-1}	1.09	0.858-1.386	7.8×10^{-1}	0.87	0.655-1.157	9.7×10^{-1}	1.15	0.565-2.321	2.2×10^{-1}	0.56	0.344-0.929
Pancreatic develop-ment	HHEX	rs5015480	1.9×10^{-1}	0.86	0.731-1.007	1.8×10^{-1}	0.82	0.685-0.981	8.0×10^{-1}	0.92	0.728-1.167	2.1×10^{-1}	0.69	0.521-0.915	9.7×10^{-1}	1.07	0.541-2.116	9.5×10^{-1}	0.89	0.552-1.442
	IGF2BP2	rs4402960	4.8×10^{-1}	1.11	0.933-1.312	2.6×10^{-1}	1.20	0.985-1.451	5.0×10^{-1}	1.32	1.025-1.711	9.4×10^{-1}	1.04	0.769-1.414	9.7×10^{-1}	0.99	0.482-2.030	9.5×10^{-1}	0.83	0.523-1.328
Insulin resistance	GCKR	rs780094	1.8×10^{-1}	1.25	0.647-0.993	4.0×10^{-1}	1.18	0.671-1.078	5.5×10^{-1}	1.32	0.563-1.024	9.1×10^{-1}	0.91	0.725-1.655	9.7×10^{-1}	2.22	0.156-1.316	9.5×10^{-1}	1.19	0.424-1.657
	KLF14	rs972283	9.4×10^{-1}	1.02	0.808-1.278	6.8×10^{-1}	0.92	0.705-1.193	9.0×10^{-1}	0.93	0.666-1.298	8.6×10^{-1}	0.87	0.549-1.368	9.7×10^{-1}	1.77	0.629-5.005	9.5×10^{-1}	1.26	0.639-2.469
	PPARG	rs1801282	8.3×10^{-1}	0.96	0.685-1.341	7.3×10^{-1}	0.93	0.640-1.340	9.9×10^{-1}	1.04	0.604-1.774	7.8×10^{-1}	0.78	0.488-1.360	9.7×10^{-1}	2.41	0.255-22.710	9.5×10^{-1}	0.86	0.331-2.223
		rs1373641	2.8×10^{-1}	0.87	0.730-1.039	2.5×10^{-1}	0.82	0.674-1.007	7.6×10^{-1}	0.89	0.681-1.167	4.8×10^{-1}	0.75	0.555-1.020	9.7×10^{-1}	1.39	0.611-3.141	9.5×10^{-1}	0.97	0.617-1.522
		rs709149	1.4×10^{-1}	0.81	0.684-0.956	1.8×10^{-1}	0.78	0.648-0.944	5.5×10^{-1}	0.83	0.643-1.074	3.4×10^{-1}	0.73	0.553-0.972	9.7×10^{-1}	0.91	0.441-1.866	9.5×10^{-1}	0.88	0.555-1.385

S1 Table. Type 2 Diabetes Risk Allele SNPs (cont., page 3)

			All Qatari			Q1 +Q2[1]			Q1[1]			Q2[1]			Q3[1]			Admixed[1]		
**Category**	**Gene**	**SNP**	**p value[2]**	**OR[3]**	**CI[4]**	**p value[2]**	**OR[3]**	**CI[4]**	**p value[2]**	**OR[3]**	**CI[4]**	**p value[2]**	**OR[3]**	**CI[4]**	**p value[2]**	**OR[3]**	**CI[4]**	**p value[2]**	**OR[3]**	**CI[4]**
Possible insulin resistance	ADAMTS9	rs4607103	7.9×10^{-1}	1.05	0.892-1.243	5.8×10^{-1}	1.08	0.899-1.306	9.0×10^{-1}	1.05	0.827-1.344	7.9×10^{-1}	1.14	0.845-1.525	9.7×10^{-1}	1.12	0.601-2.100	9.5×10^{-1}	1.09	0.831-2.313
Obesity	FTO[5]	rs1075440	8.1×10^{-1}	0.96	0.784-1.176	6.9×10^{-1}	0.94	0.750-1.168	7.6×10^{-1}	0.89	0.652-1.208	9.8×10^{-1}	0.99	0.723-1.372	9.7×10^{-1}	0.95	0.344-2.628	9.5×10^{-1}	1.16	0.627-2.157
		rs13333228	7.9×10^{-1}	0.94	0.740-1.196	9.3×10^{-1}	0.99	0.758-1.282	9.7×10^{-1}	1.03	0.717-1.489	9.1×10^{-1}	0.93	0.628-1.370	9.7×10^{-1}	0.50	0.136-1.864	9.5×10^{-1}	0.79	0.381-1.621
		rs8047395	7.9×10^{-1}	0.96	0.814-1.130	7.4×10^{-1}	0.96	0.801-1.151	9.1×10^{-1}	0.95	0.747-1.216	9.7×10^{-1}	0.98	0.746-1.296	9.7×10^{-1}	0.59	0.288-1.224	9.5×10^{-1}	1.06	0.657-1.695
		rs9930333	8.4×10^{-1}	0.98	0.821-1.159	9.1×10^{-1}	0.99	0.812-1.196	9.9×10^{-1}	0.99	0.760-1.296	9.7×10^{-1}	1.02	0.760-1.358	9.7×10^{-1}	1.11	0.586-2.107	9.5×10^{-1}	1.22	0.743-2.015
		rs17817449	7.9×10^{-1}	1.05	0.893-1.233	7.4×10^{-1}	1.04	0.870-1.249	8.2×10^{-1}	0.92	0.725-1.178	9.7×10^{-1}	0.99	0.753-1.302	9.7×10^{-1}	1.10	0.598-2.033	9.5×10^{-1}	1.20	0.742-1.934
		rs8050136	8.1×10^{-1}	1.04	0.881-1.218	7.5×10^{-1}	1.04	0.864-1.240	9.2×10^{-1}	0.96	0.751-1.219	9.7×10^{-1}	1.02	0.776-1.345	9.7×10^{-1}	1.25	0.649-2.390	9.5×10^{-1}	1.11	0.688-1.790
		rs9939609	8.1×10^{-1}	1.03	0.878-1.216	6.9×10^{-1}	0.95	0.789-1.139	9.6×10^{-1}	0.97	0.761-1.244	8.6×10^{-1}	1.09	0.824-1.443	9.7×10^{-1}	0.95	0.502-1.814	9.5×10^{-1}	1.10	0.683-1.760
		rs7201850	7.9×10^{-1}	1.08	0.804-1.442	7.4×10^{-1}	0.93	0.662-1.302	7.6×10^{-1}	1.21	0.737-1.987	9.7×10^{-1}	1.05	0.637-1.715	9.7×10^{-1}	1.05	0.316-3.459	9.5×10^{-1}	1.08	0.448-2.613
		rs11642841	2.4×10^{-1}	1.16	0.981-1.363	2.5×10^{-1}	1.18	0.989-1.417	6.3×10^{-1}	1.14	0.897-1.436	4.8×10^{-1}	1.28	0.962-1.695	9.7×10^{-1}	0.61	0.260-1.447	9.5×10^{-1}	1.30	0.790-2.136
		rs11075995	3.5×10^{-1}	1.12	0.950-1.328	4.0×10^{-1}	1.14	0.943-1.370	5.8×10^{-1}	1.16	0.901-1.480	8.0×10^{-1}	1.12	0.841-1.487	9.7×10^{-1}	1.36	0.610-3.039	9.5×10^{-1}	0.97	0.616-1.532
Circadian rhythm	CRY2	rs11605924	5.0×10^{-1}	0.89	0.720-1.098	4.8×10^{-1}	0.87	0.682-1.101	5.5×10^{-1}	0.73	0.536-0.993	7.8×10^{-1}	1.25	0.834-1.873	9.7×10^{-1}	1.27	0.465-3.484	9.5×10^{-1}	1.11	0.605-2.018

[1] Q1=Bedouin, Q2=Persian/South Asian, Q3=African, Admixed=Structure cut-off <0.65 in all sub-populations (k=3).
[2] Benjamini-Hochberg corrected p-value.
[3] Estimated odds ratio (OR).
[4] Lower and upper bounds of 95% confidence interval (CI) for odds ratio.
[5] No BMI covariate used for FTO gene.
