# Supplementary material for: Type 2 Diabetes Risk Allele Loci in the Qatari Population
Source: PLoS One. 2016 Jul 6;11(7):e0156834. doi: 10.1371/journal.pone.0156834 (PMC4934876; doi:10.1371/journal.pone.0156834)
Supplement: S2 Table — (PDF) [file pone.0156834.s004.pdf]

S2 Table. Statistical Power to Detect Associations with Type 2 Diabetes for Known Risk Alleles

Gene	SNP	Risk allele	Cases	Con-trols	OR[1]	Power	Cases for 80% power[2]	Power at max OR=2.71[3]	Cases for 80% power at max OR=2.71[3]	Mini-mum OR for 80% power[4]	Refer-ence
CAMK1D/CDC123	rs12779790	G	1067	554	1.11	0.27	4652	1	71	1.24	[1]
CDKAL1	rs10946398	C	982	519	1.12	0.41	2605	1	50	1.20	[2]
	rs7756992	G	624	326	1.20	0.61	970	1	50	1.26	[3]
CDKN2A-B	rs564398	T	1108	576	1.12	0.35	3547	1	89	1.23	[2]
	rs10811661	T	1071	557	1.20	0.64	1572	1	107	1.26	[4]
G6PC2	rs560887	G	560	300	1.03	0.06	51441	1	91	1.34	[5]
HHEX/IDE/KIF11	rs1111875	C	1021	526	1.19	0.78	1068	1	63	1.20	[6]
HNF1A	rs7957197	T	546	296	1.07	0.10	10951	1	95	1.35	[7]
HNF1B (TCF2)	rs4430796	G	620	325	1.11	0.26	2752	1	54	1.25	[7]
JAZF1	rs864745	T	1071	564	1.10	0.37	3190	1	53	1.19	[1]
KCNJ11	rs5215	C	1089	565	1.14	0.49	2294	1	58	1.21	[2]
MADD	rs7944584	A	623	325	1.01	0.05	418201	1	85	1.31	[5]
NOTCH2	rs10923931	T	1087	576	1.13	0.30	4174	1	86	1.27	[1]
THADA	rs7578597	T	1117	588	1.15	0.28	4650	1	192	1.34	[1]
ADRA2A	rs10885122	G	622	323	1.04	0.07	25174	1	74	1.29	[5]
FADS1	rs174550	T	624	325	1.04	0.07	23972	1	70	1.28	[5]
GCK	rs1799884	A	617	321	1.34	0.95	382	1	52	1.27	[8]
	rs4607517	A	621	319	1.07	0.12	8019	1	53	1.27	[5]
GLIS3	rs7034200	A	619	324	1.27	0.86	527	1	54	1.25	[9]
LGR5/TSPAN8	rs7961581	C	1090	579	1.09	0.31	3931	1	47	1.18	[1]
PROX1	rs340874	C	623	325	1.07	0.14	6524	1	47	1.24	[5]
SLC2A2(GLUT2)	rs11920090	T	625	325	1.01	0.05	509320	1	107	1.34	[5]
SLC30A8	rs11558471	A	621	323	2.17	1.00	120	1	120	1.37	[10]
	rs13266634	C	437	332	1.18	0.31	1629	1	95	1.39	[11]
TCF7L2	rs4506565	T	1033	547	1.36	1.00	297	1	47	1.19	[12]
	rs7901695	C	1107	584	1.37	1.00	281	1	47	1.18	[2]
	rs7903146	T	1079	571	1.54	1.00	153	1	47	1.18	[13]
HHEX	rs5015480	C	1073	546	1.16	0.71	1320	1	55	1.19	[7]
IGF2BP2	rs4402960	T	1075	533	1.14	0.59	1763	1	49	1.19	[2]
GCKR	rs780094	C	619	322	1.04	0.09	15425	1	57	1.26	[14]
KLF14	rs972283	G	518	295	1.07	0.12	6671	1	53	1.27	[7]
PPARG	rs1801282	C	1105	566	1.14	0.19	7647	1	289	1.44	[4]
ADAMTS9	rs4607103	C	1031	531	1.09	0.32	3692	1	48	1.19	[1]
FTO	rs11642841	A	1098	567	1.12	0.49	2305	1	47	1.18	[7]

S2 Table. Statistical Power to Detect Associations with Type 2 Diabetes for known Risk Alleles (cont., page 2)

Gene	SNP	Risk allele	Cases	Controls	OR[1]	Power	Cases for 80% power[2]	Power at max OR=2.71[3]	Cases for 80% power at max OR=2.71[3]	Minimum OR for 80% power[4]	Reference
	rs8050136	A	1109	567	1.17	0.79	1142	1	49	1.18	[2]
	rs9939609	A	1077	553	1.34	1.00	329	1	49	1.18	[12]
CRY	rs11605924	A	626	326	1.14	0.41	1624	1	49	1.24	[9]

1 Odds ratio.

2 Based on published odds ratios (OR).

3 Based on maximum odds ratios (OR) of 37 T2D associated SNPs.

4 Minimum odds ratio (OR) with >80% power to detect an association given the sample size; calculated using a range of OR between 1.01 and 2.17, in 0.01 step intervals
