# Supplementary material for: Type 2 Diabetes Risk Allele Loci in the Qatari Population
Source: PLoS One. 2016 Jul 6;11(7):e0156834. doi: 10.1371/journal.pone.0156834 (PMC4934876; doi:10.1371/journal.pone.0156834)
Supplement: S3 Table — (PDF) [file pone.0156834.s005.pdf]

S3 Table. Demographics of Type 2 Diabetic Cases and Controls[1]

Parameters	European SNPs			South Asian SNPs		
	Controls	T2D cases	p value	Controls	T2D cases	p value
n	590	1124		326	626	
Gender (M/F)[4]	213/377	447/677	$1.4x10^{-1}$	68/258	201/425	$2.5x10^{-4}$
Age at examination (yr) [1,5]	46 ± 9	55 ± 10	$p<10^{-10}$	46 ± 9	55 ± 10	$p<10^{-10}$
Genetic subpopulation[2,4] (Q1/Q2/Q3/ Admixed)[3]	46.4/34.6/ 6.3/12.7	44.9/33.0/ 8.5/13.5	$9.4x10^{-1}$	49.4/31.0/ 5.5/14.1	46.2/29.9/ 9.9/14.1	$7.1x10^{-1}$
BMI (kg/m^2)[1,5]	32 ± 7	34 ± 7	$1.4x10^{-6}$	32 ± 8	34 ± 7	$8.3x10^{-4}$
HbA1c (%)[1,5,6]	5.6 ± 0.4	8.3 ± 1.9	$p<10^{-10}$	5.6 ± 0.4	8.4 ± 2.0	$p<10^{-10}$
Creatinine (µmol/L) [1,5]	72.6 ± 29.7	81.5 ± 71.5	$1.5x10^{-3}$	70.1 ± 34.1	79.4 ± 73.4	$5.6x10^{-2}$
Glucose (mmol/L) [1,5]	5.2 ± 0.8	9.5 ± 4.6	$p<10^{-10}$	5.1 ± 0.7	9.4 ± 4.9	$p<10^{-10}$

[1] Data are presented as mean ± standard deviation.
[2] Data are presented as percent of total n.
[3] Q1=Bedouin, Q2=Persian/South Asian, Q3=African, Admixed=Structure cut-off <0.65 in all sub-populations (k=3).
[4] Chi-square p value.
[5] Student's t-test two-tailed distribution with unequal variance p value.
[6] HbA1C level available for 336 control subjects.
[7] Glucose level available for 284 control subjects.
